# Reactive agility—evidence based suggestions for improvement

**DOI:** 10.3389/fspor.2026.1809831

**Published:** 2026-04-15

**Authors:** Lutz Vogt, Felix Laukhardt, Christian Haser, Winfried Banzer, Tobias Engeroff

**Affiliations:** 1 Department of Sports Medicine & Exercise Physiology, Goethe University Frankfurt, Frankfurt, Germany; 2 Institute of Occupational, Social and Environmental Medicine, Goethe University Frankfurt, Frankfurt, Germany; 3 Medical Department, Eintracht Frankfurt Fußball AG, Frankfurt, Germany

**Keywords:** change of direction (COD), lower-limb muscular capacities, neuromuscular–cognitive interaction, perceptual–cognitive performance, reactive agility

## Abstract

**Purpose:**

To examine the contributions of lower-limb eccentric strength, reactive strength, and cognitive inhibitory control to reactive agility performance.

**Methods:**

Forty-five healthy, physically active males (21.3 ± 3.5 years) performed eccentric and isometric strength testing of knee flexors and extensors (Biodex dynamometer), 30 cm drop jumps for reactive strength, and a computerized Stroop task (EncephalApp) for cognitive performance. Reactive agility was assessed via the Random Star Run (RSR) on the SKILLCOURT. Multiple linear regression analyzed associations of strength and cognitive performance outcomes with reactive agility.

**Results:**

Mean eccentric strength of the knee flexors (EccHam/kg) and extensors were 2.24 ± 0.44 and 4.07 ± 0.79 Nm·kg^−^^1^; isometric strength was 1.71 ± 0.285 Nm·kg^−^^1^ and 3.79 ± 0.751 Nm·kg^−^^1^; RSR duration was 17.03 ± 1.36 s; Stroop Interference Score was 1.01 ± 1.07 s; Drop Jump height averaged 0.328 ± 0.058 m and ground contact time (DJ GCT) was 0.189 ± 0.024 s. Higher EccHam/kg, lower Stroop interference, and shorter DJ GCT predicted faster RSR performance (*R*^2^ = .338, *p* < 0.05). Thirteen participants exceeded the upper tertile on at least two variables (EccHam/kg ≥ 2.5 Nm·kg^−^^1^, Stroop ≤ 0.415 s, DJ GCT ≤ 0.174 s) and showed significantly faster RSR times and roughly doubled likelihood of above-average reactive agility performance (RSR ≤ 17 s; RR = 2.04, 95% CI = 1.17–3.54).

**Conclusions:**

Reactive agility is enhanced by higher eccentric hamstring strength, superior cognitive inhibitory control, and fast stretch–shortening cycle execution. The identified cut-offs may serve as practical benchmarks for training programs targeting eccentric strength, cognitive control, and plyometric efficiency. These findings underscore the combined neuromuscular and cognitive determinants of reactive agility, warranting longitudinal studies to confirm causality.

## Introduction

Reactive agility, defined as the ability to quickly and accurately respond to unpredictable external stimuli with rapid whole-body movements, including direction changes, plays a crucial role in many open-skill sports (1). Unlike traditional change-of-direction (COD) tasks, which are usually pre-planned and closed-skill without reactive demands, reactive agility involves complex cognitive-perceptual processes such as visual scanning and decision-making alongside neuromuscular execution (2).

Lower-limb muscular capacities are considered key determinants of COD and agility performance (1, 3). High eccentric force capacity appears to facilitate effective deceleration and upper-body stabilization during rapid directional changes (4), whereas high maximal isometric torque is reported to have an influence on COD performance (5) and could be a crucial factor for initiating subsequent re-acceleration. Moreover, the interplay between muscular coordination and maximal force production—evidenced by short ground contact times in the stretch–shortening cycle—could be essential for effective movement by enabling efficient force absorption and reapplication during deceleration and acceleration (1). However, these biomechanical insights mainly derive from closed, pre-planned COD tasks and therefore might capture only a limited part of reactive agility performance.

According to Sheppard and Young (1), reactive agility also relies on athletes' capacity to perceive, interpret, and respond to unpredictable stimuli, making perceptual–cognitive processes a vital component of successful performance. Building on this, research increasingly highlights the role of visual processing speed and executive functions, which may even outweigh muscular strength under ecologically valid, unpredictable conditions (2). Simultaneously, the growing use of reactive agility tools in sports and rehabilitation highlights the need to evaluate their relevance for diagnostics and training. Validated systems such as SKILLCOURT offer structured ways to assess reactive agility performance (6, 7).

This cross-sectional study examines how these neuromuscular and cognitive factors—and their interactions—relate to reactive agility and to identify performance thresholds that could improve understanding of underlying mechanisms and inform targeted neuromuscular–cognitive training in both athletic and clinical contexts.

## Methods

### Participants

Forty-five healthy male subjects (age 21.3 ± 3.52 years; height 182 ± 6.55 cm; body mass 78.1 ± 8.05 kg) volunteered to participate in this study. All participants had several years of experience in team sports involving reactive agility demands and reported participating in at least one team sport training session per week. Nearly half of the sample were actively competing at the time of testing. All participants were free from musculoskeletal, cardiovascular, and neurological disorders. Prior to study enrollment, all participants provided written informed consent. The study was conducted in accordance with the Declaration of Helsinki and approved by the local institutional ethics committee (number 2024-30). Participants were informed about the study procedures, potential risks, and their right to withdraw at any time.

### Agility assessment

Reactive agility was assessed using the Random Star Run on the SKILLCOURT (movement concepts GmbH, Schweinfurt, Germany). The system consists of a 65″ monitor positioned in front of a 500 cm × 500 cm court containing one center field and eight evenly spaced square target fields arranged radially around the center. Participant movements were tracked using a light imaging, detection and ranging system.

At the beginning of the test, participants stood on the center field. A visual stimulus was presented on the monitor displaying a graphical representation of the court and indicating the next target field. Participants were required to react as quickly as possible by moving from the center to the indicated target field. After reaching the target field, participants returned to the center position, where the next stimulus was presented. The sequence of target locations was randomized to prevent anticipation. Each trial consisted of 8 choice reaction stimuli (target fields) and 8 pre-planned stimuli (center field), and the fastest completion time out of two trials was used for analysis.

The task therefore required rapid perception of the visual stimulus and a reactive change of direction toward the indicated target location. No additional distractors or inhibitory stimuli were included. For subsequent analysis, the fastest completion time (s) of three recorded trials was used. Before formal testing, participants completed a familiarization trial on the SKILLCOURT approximately 1 week before the actual assessment to reduce learning effects and ensure consistent performance. This session allowed practice of the task and was not included in the analysis (6).

### Strength assessment

Eccentric and isometric maximal strength of the knee flexors and extensors of one randomly selected extremity was measured using a Biodex dynamometer (Biodex Medical Systems, Shirley, NY, USA) to avoid systematic bias related to limb dominance. Participants were seated with the hip and knee joints initially positioned at 90°. The dynamometer axis was aligned with the lateral femoral epicondyle. Participants were stabilized using straps across the trunk, pelvis, and thigh to minimize compensatory movements. For eccentric testing, contractions were performed through a knee joint range of motion from 90° to 10° of knee flexion at an angular velocity of 60° s^−^^1^. This relatively low angular velocity was selected to allow the assessment of maximal eccentric torque under controlled conditions and to obtain reliable peak torque values using isokinetic dynamometry. For isometric testing, maximal voluntary contractions were performed at a fixed knee joint angle of 90° knee flexion. Following a standardized warm-up, participants performed 8 s isometric contractions (for flexors and extensors) and 6 eccentric repetitions per muscle group, with the highest value used for analysis. The peak torque was normalized to body weight (Nm·kg^−^^1^). Standardized verbal encouragement was provided during the assessment to ensure maximal effort and enhance motivation.

### Reactive strength testing

Reactive strength was assessed using a 30 cm drop jump on a contact mat (Refitronic, Schmitten, Germany) to evaluate stretch–shortening cycle performance. Participants stepped off the platform, landed on both feet, and immediately performed a maximal vertical jump while minimizing ground contact time. Participants performed 3 trials, with the best trial used for analysis. Ground contact time (s), jump height (m) and reactive strength index (RSI) were assessed for further analysis. Trials with contact times longer than 250 ms were considered technique-faulty and excluded from further analysis (8).

### Executive cognitive function assessment

Cognitive interference was assessed using a computerized Stroop task implemented with EncephalApp—Stroop Test for iOS is 2.2.0 (9). Participants completed both congruent (“OFF”) and incongruent (“ON”) trials, in which they were required to identify the color of the stimulus while ignoring the word itself. The application reports the total time required to complete a sequence of ten stimuli within each condition, rather than the reaction time to a single stimulus. The Stroop interference score was calculated as the difference between incongruent and congruent trial completion times.

### Procedures and analysis

Reactive agility, strength, drop-jumps and executive function testing were performed on separate days, with approximately 1 week between sessions. Statistical analyses were conducted using *IBM SPSS Statistics (Version 31)*. Descriptive statistics are presented as mean ± standard deviation (SD).

Determinants of reactive agility performance were analyzed using multiple linear regression analysis with Random Star Run duration (RSR duration) as the dependent variable. Eccentric and isometric hamstring and quadriceps strength normalized to body mass; Stroop On and Off performance as well as interference score; drop jump ground contact time, jump height, and the reactive strength index (RSI) were initially considered as potential predictor variables.

Given the exploratory aim of the study and the relatively small sample size, a stepwise multiple linear regression procedure was applied to identify the subset of variables that best explained the variance in RSR duration. Prior to interpretation of the regression model, standard assumptions of linear regression, including linearity, normal distribution of residuals, homoscedasticity and absence of multicollinearity (variance inflation factors, VIF), were inspected. The level of statistical significance was set at *α* = 0.05.

## Results

The mean relative eccentric strength of the knee flexors (*EccHam/kg*) and extensors (*EccQuad/kg*) was 2.24 ± 0.44 Nm·kg^−^^1^ and 4.07 ± 0.79 Nm·kg^−^^1^, respectively. The mean relative isometric strength of the knee flexors (*IsomHam/kg*) and extensors (*IsomQuad/kg*) was 1.71 ± 0.285 Nm·kg^−^^1^ and 3.79 ± 0.751 Nm·kg^−^^1^. The mean completion time for the *Random Star Run (RSR duration)* was 17.03 ± 1.36 s. Mean *Stroop ON* and *Stroop OFF* times were 11.3 ± 1.64 s (1.13 ± 0.16 s per stimulus) and 10.3 ± 1.01 s (1.03 ± 0.10 s per stimulus), resulting in a mean *Stroop Interference Score* of 1.01 ± 1.07 s (0.10 ± 0.11 s per stimulus). The average *Drop Jump ground contact time (DJ GCT)* was 0.189 ± 0.024 s, *Drop Jump height (DJ height)* averaged 0.328 ± 0.058 m and *Reactive Strength Index (RSI)* was 1.76 (SD = 0.39).

Stepwise multiple linear regression analysis identified eccentric hamstring strength normalized to body mass (EccHam/kg), Stroop interference score and drop jump ground contact time (DJ GCT) as significant predictors of Random Star Run duration. The final regression model demonstrated a moderate model fit (*R* = 0.581, *R*^2^ = 0.338, *N* = 44), explaining 33.8% of the variance in reactive agility performance. The model predicted RSR duration with a mean absolute error (MAE) of 0.793 s and a root mean squared error (RMSE) of 1.03 s, corresponding to approximately 4.7% of the observed mean RSR duration (17.03 s). No relevant multicollinearity was detected among the predictors (VIF = 1.004–1.005).

Eccentric hamstring strength showed a significant negative association with RSR duration (*B* = −0.942, *β* = −0.342, *p* = 0.016), indicating that greater eccentric hamstring strength was associated with faster completion times. In contrast, Stroop interference (*B* = 0.483, *β* = 0.432, *p* = 0.003) and drop jump ground contact time (*B* = 14.365, *β* = 0.270, *p* = 0.047) were positively associated with RSR duration, suggesting that greater cognitive interference and longer ground contact times were related to slower reactive agility performance. All other candidate variables were excluded during the stepwise selection procedure and did not contribute significantly to the final regression model.

**Figure 1:**
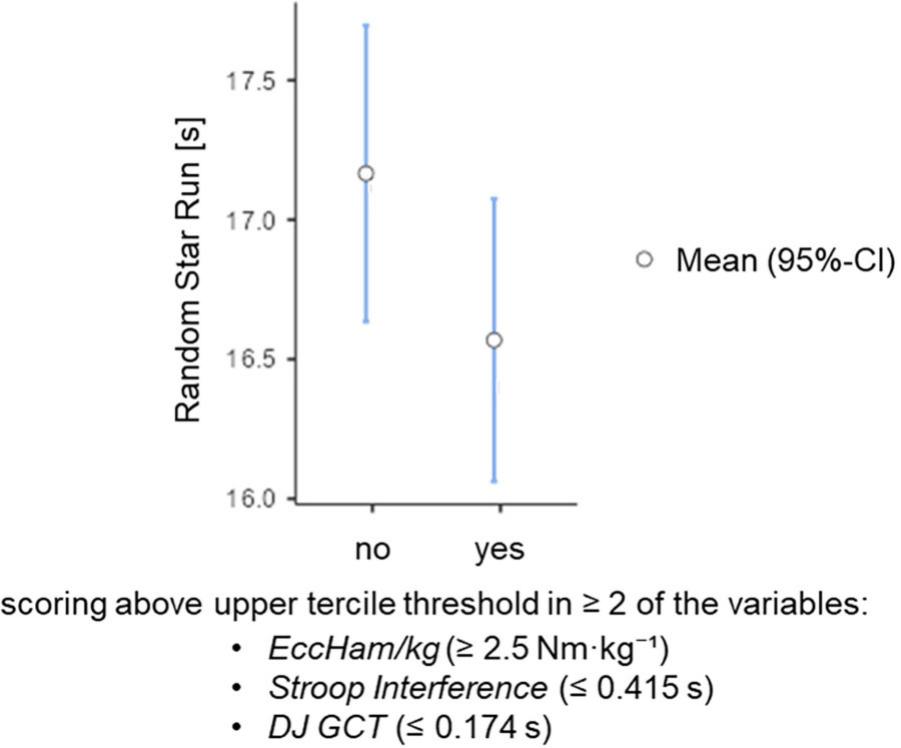
Comparison of RSR duration between participants who scored in the upper tertile on at least two of the three variables (relative eccentric hamstring strength, Stroop interference, and drop jump ground contact time) vs. those who did not.

Tertile-based subgroup analysis for the variables detected as relevant in the multiple regression revealed that a total of thirteen participants were positioned in the upper tertile on at least two of the three variables (*EccHam/kg* ≥ 2.5 Nm·kg^−^^1^, *Stroop Interference* ≤ 0.415 s, *DJ GCT* ≤ 0.174 s). Of these subjects, one scored in all three variables, five in *Stroop Interference* and *EccHam/kg*, four in *Stroop Interference* and *DJ GCT*, and three in *EccHam/kg* and *DJ GCT*. Participants scoring in the upper tertile on at least two of the three variables exhibited significantly lower RSR values (*p* < .05; Figure 1) and had approximately twice the likelihood (RR = 2.04; 95% CI: 1.17–3.54) of achieving above-average RSR performance (*RSR duration* ≤ 17 s).

## Discussion

The present findings demonstrate significant associations of eccentric maximal peak torque of the hamstrings, drop jump ground contact time and cognitive interference control with reactive agility performance. Based on the exploratory tertile-based comparisons, our data further suggest that different combinations of enhanced knee deceleration control and stabilization, faster executive control performance, and a faster stretch–shortening cycle may support faster execution of a lower-extremity choice-reaction task. Due to the exploratory nature of the regression analysis, the limited sample size, and the additional use of cut-off based comparisons, the results should be interpreted with caution and require confirmation in larger samples.

Our strength testing data confirm the pivotal role of eccentric force production in effective deceleration and whole-body stabilization during rapid directional changes (4, 10). In addition, our data extend current evidence by (1) demonstrating the comparatively greater relevance of hamstring eccentric capacity compared to quadriceps performance for reactive agility and (2) by showing that eccentric strength remains influential within a reactive agility paradigm with multiple changes of direction in response to visual stimuli. Whereas multiple earlier studies already reported the relevance of explosive strength based on a counter movement jump (4, 11, 12) our study differentiates explosive power and reactive strength by using drop jumps and indicates that reactive strength and therefore neuromuscular reaction time is more important than peak torque during the concentric phase of the jump.

A large number of studies have already confirmed the impact of cognitive performance on reactive agility (2, 12). Some studies even highlight that for reactive agility tasks consisting of a single movement initiation to a visual stimulus strength prerequisites or linear acceleration performance are irrelevant compared to their value for COD performance (2, 4). Our findings reinforce the assumption that the capability to perform higher cognitive tasks, such as executive functions, is more relevant for reactive agility performance than processing speed for lower cognitive functions (2, 12). Furthermore, our data underlines that performance during a task consisting of multiple changes of direction in response to visual stimuli is related to both processing of visual stimuli and strength performance (12) and generates a more detailed picture by detecting that both reactive strength and eccentric strength are important capabilities.

These findings provide practical guidance for designing training programs to enhance reactive agility. Existing literature highlights the importance of eccentric strength training for improving COD performance (3, 10), and our results support this emphasis. In addition, our data suggest that targeted strengthening of key muscle groups and the integration of plyometric exercises (13) may offer additional benefits specifically for improving reactive agility. From a practical training perspective, eccentric hamstring strength values around 2.5 Nm·kg^−^^1^ [right between the average eccentric strength levels reported for elite and professional soccer players ∼2.39 vs. ∼2.74 Nm·kg^−^^1^ (14); or professional soccer players according to their field position ∼2.51–2.79 Nm·kg^−^^1^ (15)] and drop jump ground contact times around 0.174 s [slightly below a recently recommended <0.188 s threshold for fast stretch–shortening cycle actions (16)] may serve as descriptive reference points. However, these values were derived from exploratory cut-off based analyses and should therefore not be interpreted as definitive performance thresholds.

Instead of training abstract cognitive tasks that resemble traditional cognitive performance tests, current research suggests that it is more effective to combine realistic stimuli with single-movement initiations to train cognitive-perceptual skills (17). Our findings highlight the relevance of perceptual–motor processing and rapid stimulus–response mapping for reactive agility performance. However, tasks that incorporate additional cognitive demands, such as inhibition or interference, may further challenge the cognitive–motor system and should therefore be considered in future training and testing paradigms. In practical applications, agility training may benefit not only from exercises that emphasize rapid action initiation but also from tasks that require athletes to modify or inhibit pre-planned responses.

## Practical application

This study shows that reactive agility performance is jointly shaped by eccentric hamstring strength, reactive strength, and higher-order cognitive control. Eccentric force production and a rapid stretch–shortening cycle emerged as key physical determinants, while executive functions—particularly interference control—proved more relevant than basic processing speed. These findings highlight that effective training should integrate eccentric and plyometric strength development with realistic, visually driven decision-making tasks that require not only action initiation but also inhibition and rapid response modification.

## Data Availability

The raw data supporting the conclusions of this article will be made available by the authors, without undue reservation.
